# Associations between dimensions of mental health literacy and adolescent help‐seeking intentions

**DOI:** 10.1111/camh.12608

**Published:** 2022-11-14

**Authors:** Claire Goodfellow, Anna Macintyre, Lee Knifton, Edward Sosu

**Affiliations:** ^1^ MRC / CSO Social and Public Health Sciences Unit University of Glasgow Glasgow UK; ^2^ Centre for Health Policy University of Strathclyde Glasgow UK; ^3^ School of Social Work and Social Policy University of Strathclyde Glasgow UK; ^4^ Mental Health Foundation Glasgow UK; ^5^ School of Education University of Strathclyde Glasgow UK

**Keywords:** Adolescence, mental health literacy, help‐seeking, structural equation modelling

## Abstract

**Background:**

The majority of long‐term mental health problems begin during adolescence. Low mental health literacy (MHL) may impede help‐seeking for these problems. Although MHL is a multidimensional construct and adolescent help‐seeking can be through formal and informal means, little is known about how dimensions of MHL influence these help‐seeking intentions. This study examines associations between dimensions of MHL and formal and informal help‐seeking intentions among adolescents. It also investigates whether informal help‐seeking mediates the association between dimensions of MHL and formal help‐seeking, and whether these associations are moderated by gender.

**Methods:**

A cross‐sectional survey including measures of MHL, and help‐seeking intentions was distributed to participants in 10 schools (12–17 years) across Scotland (*n* = 734). Data were analysed using Confirmatory Factor Analyses (CFA) and Structural Equation Modelling (SEM).

**Results:**

Confirmatory Factor Analyses identified two distinct dimensions of MHL: ability to identify a mental health problem, and knowledge of treatment efficacy. Only knowledge of treatment efficacy was associated with increased intention to seek formal and informal help. Ability to identify a mental health problem was negatively associated with both forms of help‐seeking intentions. Informal help‐seeking mediated the association between both forms of MHL and formal help‐seeking. Gender did not moderate the associations between MHL and help‐seeking.

**Conclusions:**

Care should be taken when providing MHL interventions to ensure that adaptive forms of MHL are promoted. Future research should investigate possible mechanisms by which discrete forms of MHL influence adolescent help‐seeking as well as investigating other potential moderators of MHL and help‐seeking, such as stigma.

**Key practitioner message**

- We investigated how mental health literacy (MHL) impacts on formal and informal help‐seeking intentions among adolescents.
- We identified two distinct dimensions of MHL: ability to identify a mental health problem, and knowledge of treatment efficacy.
- Increased knowledge of treatment efficacy was associated with increased formal and informal help‐seeking intentions whereas increased ability to identify specific mental health problems was associated with decreased formal and informal help‐seeking intentions.
- Effective MHL interventions for adolescents should focus on providing information relating to effective treatments of mental health problems, which was associated with increased help‐seeking intentions.

## Introduction

Adolescence is a particularly vulnerable period for the development of mental health problems, with approximately half of all problems emerging before the age of 14 (Kessler et al., 2005). Crucially, mental health problems during this period can have a significant impact on health, educational and socioeconomic outcomes across the life course (Kieling et al., 2011). However, the majority of young people do not seek help for their problems (Polanczyk, Salum, Sugaya, Caye, & Rohde, 2015). For instance, in a study of adolescents in the United States, Merikangas et al. (2011) found that nearly 64% of those with clinical symptoms did not seek help from formal services. This is concerning given that a study among UK adolescents found that those who do not seek help are seven times more likely to see a worsening of mental health problems than those who do seek help (Neufeld, Dunn, Jones, Croudance, & Goodyear, 2017).

While there are multiple factors which may inhibit help‐seeking, such as social stigma, limited access to services, or negative perceptions of the therapeutic relationship, a lack of mental health knowledge is frequently noted as a significant barrier to effective help‐seeking (Radez et al., 2021). Indeed, adolescents are known to have low levels of mental health literacy (MHL; Myers et al., 2009; Olsson & Kennedy, 2010). For instance, in a UK survey of 2702 young people (under 25 years) over half of those surveyed stated that they did not seek help for mental health problems because they did not understand what it was they were going through (Young Minds, 2018). To address this knowledge gap, MHL‐based interventions have been initiated around the world to encourage early help‐seeking for mental health problems among adolescents (Coburn, 2019; Rowling, 2015).

However, the evidence for MHL interventions is mixed (Ma, Anderson, & Burn, 2022; Wei, Hayden, Kutcher, Zygmunt, & McGrath, 2013). School‐based MHL interventions are based on a theory of change, whereby increased knowledge leads to behaviour change, which ultimately leads to improved mental health outcomes. However, most intervention studies do not assess for this knowledge acquisition, but rather assess only changes in outcomes (Cairns & Rossetto, 2019). As a result, it is not possible to identify whether changes in these outcomes are due to increased MHL, or which dimensions of knowledge are more, or less, effective in mediating behaviour change. As such, mechanisms of change in MHL interventions remain unclear. We argue that this lack of nuance in understanding the specific dimensions of MHL, and how they influence help‐seeking behaviours may be a key contributing factor to the mixed success of MHL‐based interventions.

Although there is a lack of clarity around the conceptualisation and measurement of MHL (Mansfield, Patalay, & Humphrey, 2020; Spiker & Hammer, 2019), it is generally seen as a multidimensional construct. MHL was originally defined as ‘knowledge and beliefs about mental disorders which aid their recognition, management or prevention’ (Jorm et al., 1997, p. 184). This definition proposed six dimensions of MHL, including the ability to recognise specific disorders, and knowledge of how to seek help. Subsequent conceptions of MHL (Kutcher, Wei, & Coniglio, 2016) propose four dimensions: how to obtain and maintain good mental health; understanding of disorders and their treatments; stigma reduction, and enhancing help‐seeking efficacy. This reflects contemporary moves towards incorporating promotion of positive mental health, and promotion of effective help‐seeking behaviours.

Despite these multidimensional conceptualisations (Kutcher et al., 2016; O'Connor, Casey, & Clough, 2014), most studies typically *measure* MHL as a unidimensional construct with a focus on knowledge of specific mental health problems (O'Connor et al., 2014). In a systematic review of MHL and its' measurement (Mansfield, Patalay, & Humphrey, 2020), only a single study aimed to identify multidimensional factors of MHL (Campos, Dias, Palha, Duarte, & Veiga, 2016). Even where studies have conceptualised and measured MHL as multidimensional, the individual dimensions are seldom explored (e.g., the MAKS; Evans‐Lacko et al., 2010). As a result, there is a dearth of understanding in the associations between domains of MHL and adolescent help‐seeking. It is likely that this absence of nuance in the conceptualisation and measurement of MHL partly explains the mixed evidence on the effectiveness of MHL‐based interventions (Wei et al., 2013).

Help‐seeking is a key dimension of MHL, and so improving MHL among adolescents is vital to promoting increased effective help‐seeking for mental health problems. Help‐seeking may be formal or informal. With regards to adolescents, formal help‐seeking (e.g., specialist mental health services) may be mediated by parents, family members or friends. This suggest that their first point of call will be these significant others who provide informal help. Indeed, it is frequently noted that adolescents prefer to seek help through such informal avenues in the first instance (Del Mauro & Jackson Williams, 2013). Evidence suggests that a lack of help‐seeking results in worsening mental health outcomes for young people (Neufeld et al., 2017), the effects of which may still be felt in adulthood (Hazell, 2007).

Good knowledge of mental health problems has been shown to be associated with help‐seeking (Ratnayake & Hyde, 2019), and good MHL is a well‐evidenced facilitator of adolescent help‐seeking (Gulliver, Griffiths, & Christensen, 2012; Kelly, Jorm, & Wright, 2007). MHL is typically rooted in theoretical models of behaviour change such as the Theory of Planned Behaviour (TPB; Ajzen, 1991), or Knowledge‐Attitude‐Behaviour models. Central to these is that knowledge acts as a precursor of behaviour change, supplemented by other factors such as attitudes, or the perceived ease of performing such a behaviour. While knowledge is not the sole mechanism of behaviour change, it is necessary in implementing such change.

Despite the evidenced association between MHL and help‐seeking, we currently lack clarity on how different dimensions of MHL may interact with and influence help‐seeking intention. Very few studies have examined whether specific dimensions of MHL are associated with formal or informal help‐seeking, and whether the associations between domains of MHL and formal help‐seeking are mediated by informal help‐seeking.

In addressing the association between MHL and help‐seeking, we must pay attention to established gender differences. It has been demonstrated that MHL tends to be higher among girls (Haavik, Joa, Hatloy, Stain, & Langeveld, 2019). Boys have lower MHL and are less likely to think education around mental illness is important (Williams & Pow, 2007). There are also gender differences in preferred sources of help, for example, boys prefer to talk to a parent, while girls are more likely to seek help from a friend and are more willing to seek formal help (Chandra & Minkovitz, 2007). There are also evidenced gender differences in relation to the stigma towards mental health problems (Dolphin & Hennessy, 2016), and stigma is another key barrier to help‐seeking. It is important to account for these gender differences and examine whether gender moderates the association between dimensions of MHL and forms of help‐seeking.

The current study aims to fill existing research gaps by investigating associations between domains of MHL, and formal and informal help‐seeking. Additionally, we aimed to examine the extent to which informal help‐seeking mediates the association between MHL and formal help‐seeking intention among adolescents. Finally, we investigate whether gender moderates the associations between MHL and help‐seeking constructs.

## Methods

### Participants and procedure

Data for the current study were obtained from adolescents (*n* = 734) attending secondary schools in Scotland. The sample was 46.9% male. Participants were aged 12–17 years, with an average age of 14.23 years (*SD* 1.51). 88.4% of participants were White, which is in line with the ethnicity profile of Scotland (i.e., 96% White; Office for National Statistics, 2020). Descriptive statistics are in Table S1.

### Measures

The term ‘mental health problems’ was operationalised during the study, rather than ‘mental illness’ or referring to specific diagnoses. Our definition of ‘mental health problems’ is listed in the supporting information, and was created following cognitive interviewing of adolescents during piloting, allowing for a developmentally valid definition of ‘mental health problems’.

### Mental health literacy

Mental health literacy was measured using the Mental Health Knowledge Schedule (MAKS; Evans‐Lacko et al., 2010). The MAKS is a 12‐item questionnaire measuring two constructs. Six questions measure participants' mental health‐related knowledge (e.g., knowledge of treatment, recognition of mental health problems), while another six measure participants ability to recognise various mental health conditions (e.g., depression). Items are measured on a 5‐point scale (1 = ‘strongly disagree’ to 5 = ‘strongly agree’). We selected the MAKS as it was one of the well‐known MHL measures and has good retest reliability with adult samples (Wei, McGrath, Hayden, & Kutcher, 2015). However, the MAKS was originally developed for adult participants and although it has been used in previous research with adolescents, measures of internal consistency were low (Chisholm et al., 2016: Mansfield, Humphrey, & Patalay, 2020). Additionally, there is no previous systematic evaluation of the factor structure to confirm its hypothesised dimensions in adolescent samples.

To address these limitations and examine the dimensions of MHL in an adolescent sample, we undertook a confirmatory factor analysis (CFA) by testing the factor structure of the MAKS following a model generation approach (Joreskog, 1993). Analysis of the original two‐factor model of the MAKS (one relating to domains of mental health knowledge and one relating to levels of recognition of mental health conditions) indicated a poor model fit: χ^2^ (53) = 449.897, *p* < .001; RMSEA = .101 (90% Confidence Interval .093, .110); CFI = .681; TLI = .602; SRMR = .0843. Factor loadings were also low for several items.

Following Garcia, Golay, Favrod, and Bonsack (2017), we eliminated negatively worded items (6, 8 and 12) which had very low factor loadings. Additionally, we excluded two further items (1: *Most people with mental health problems want to have paid employment*, and 2: *If a friend had a mental health problem, I know what advice to give them to get professional help*) with low factor loadings. The face validity of these items as a measure of MHL was also problematic given their focus on attitudes and help‐giving. Additionally, item 5 (*people with severe mental health problems can fully recover*) had a borderline acceptable factor loading (0.319), so was removed and model fit reassessed. Item 11 (assessing whether participants recognise *drug addiction* as a mental health problem) was also removed due to low factor loadings and the model reanalysed for improved fit. A new proposed model consisting of 5‐items was a better fit to the data χ^2^ (4) = 2.663, *p* = .616; RMSEA = .000 (90% Confidence Interval .000, .046; CFI = 1.00; TLI = .999; SRMR = .0133) with clear conceptual dimensions (Figure S1).

The first factor was named *knowledge of treatment efficacy* and consists of two items (3: *Medication can be an effective treatment for people with mental health problems*, and 4: *Psychotherapy can be an effective treatment for people with mental health problems*). The second factor was named *ability to identify mental health problems* and consists of three items (*Say whether you think each condition is a type of mental health problem*: *schizophrenia*, *depression*, *bi‐polar disorder*).

### Intended help‐seeking

Intended help‐seeking was measured using the General Help Seeking Questionnaire (GHSQ; Wilson, Deane, Ciarrochi, & Rickwood, 2005). The GHSQ is a 10‐item measure, which investigates intended help‐seeking on two subscales: formal and informal. Due to the age of participants, one item assessing help‐seeking from an ‘intimate partner’ was excluded. The scale used for analysis therefore contained eight items with five measuring formal and three measuring informal help‐seeking. Items are rated from 1 (*extremely unlikely to seek help*) to 7 (*extremely likely*). The GHSQ is designed to be used with secondary school aged participants and has good internal consistency (Cronbach's alpha = .85), and test–retest reliability (.92) (Wilson et al., 2005).

A CFA examining the factorial validity of the proposed two factor GHSQ model (formal and informal intended help‐seeking) in the current study was of good model fit: χ^2^ (19) = 102.792, p < .001; RMSEA = .078 (90% Confidence Interval .063, .093); CFI = .943; TLI = .917; SRMR = .0427 (Figure S2).

### Gender

Due to noted gender differences in relation to MHL and help‐seeking, gender was included as a moderator of MHL constructs and formal and informal help‐seeking. A binary measure of gender (male/female) was included for analysis. While participants were offered the option of ‘other’ this subsample was small (*n* = 3), and therefore were excluded from all analyses.

### Covariates

Several sociodemographic characteristics associated with MHL and help‐seeking were used as covariates in the study. *Ethnicity* was based on UK census categories. Due to a small proportion of participants from minority ethnic backgrounds (*n* = 83), ethnicity was dummy coded to represent White (1) and other ethnic groups (0). *Age* was measured as age in years at time of survey completion. Family subjective socioeconomic status (SES) was measured by asking participants to respond to the question *How well off do you think your family is?* (Currie et al., 2014). Responses were originally measured on a 5‐point scale (1 ‘Very well off’, to 5 ‘not at all well off’) (Svedberg, Nygren, Staland‐Nyman, & Nyholm, 2016), but recoded as ‘Low‐Medium SES’ (0) and ‘High SES’ (1) to address skewness in participants responses. *School neighbourhood deprivation* was measured using the Scottish Index of Multiple Deprivation (SIMD) tercile (‘low’ = 1; ‘mid‐range’ = 2; ‘high’ = 3). The SIMD measures and identifies areas of poverty and inequality in Scotland (Scottish Government, 2016) and higher scores represent lower levels of deprivation.

### Statistical analysis

Data were analysed using Structural Equation Modelling (SEM). In the first stage, Confirmatory Factor Analysis was used to assess the validity of measurement constructs used within the research. Then, we tested the structural model to examine hypothesised associations between dimensions of MHL and help‐seeking intentions. Finally, multi group SEM was used to evaluate the moderating effect of gender on the associations between dimensions of MHL and help‐seeking intentions. All models were evaluated using current rules of thumb with Root Mean Square Error of Approximation (RMSEA) values < than 0.5; Tucker‐Lewis Index (TLI) and Comparative Fit Index (CFI) value of >.90; and Standardised Root Mean Square Residual (SRMR) values <.08 indicative of acceptable model fit (Chen, 2007). Results from multigroup analysis were evaluated using changes in CFI > .01, RMSEA > .015 and SRMR > .030 to indicate invariance in hypothesised associations across groups (Putnick & Bornstein, 2016). All analyses were reported using a bias‐corrected bootstrapping method with 95% confidence intervals. SEM analyses were undertaken using IBM SPSS AMOS version 27.

### Missing data

Little's test was significant (χ^2^ [5183] = 5675.126, *p* < .001), indicating that the data was not missing completely at random (MCAR). However, the percentages of missingness at item level were small (0.03%–9%) therefore, Expectation Maximisation (EM: Graham, 2009) was used to impute missing data, as is recommended when data is MAR, or when the percentage of missing data is small (Allison, 2001). This is known to be similarly accurate to imputation via chained equations (Lin, 2010).

The methods and results reported in this article were part of a larger study which also collected data relating to adolescents' levels of personal and perceived stigma towards mental health problems. For an overview of the study as a whole, please refer to our previously published research (Goodfellow, Sosu, Macintyre & Knifton, 2021; Goodfellow, 2020).

## Results

### Descriptive results

Descriptive findings (Table S1 & S2) indicate high levels of MHL. Overall, adolescents reported high levels of knowledge of treatment efficacy (*M* = 7.58; *SD* = 1.55) and ability to recognise specific mental health problems (*M* = 13.40; *SD* = 1.98). Mean scores for forms of help‐seeking intention show that adolescents were more likely to use informal sources of help (*M* = 4.79, *SD* = 1.37) than formal sources (*M* = 3.11, *SD* = 1.28).

### Associations between dimensions of MHL and forms of help‐seeking

**Figure 1:**
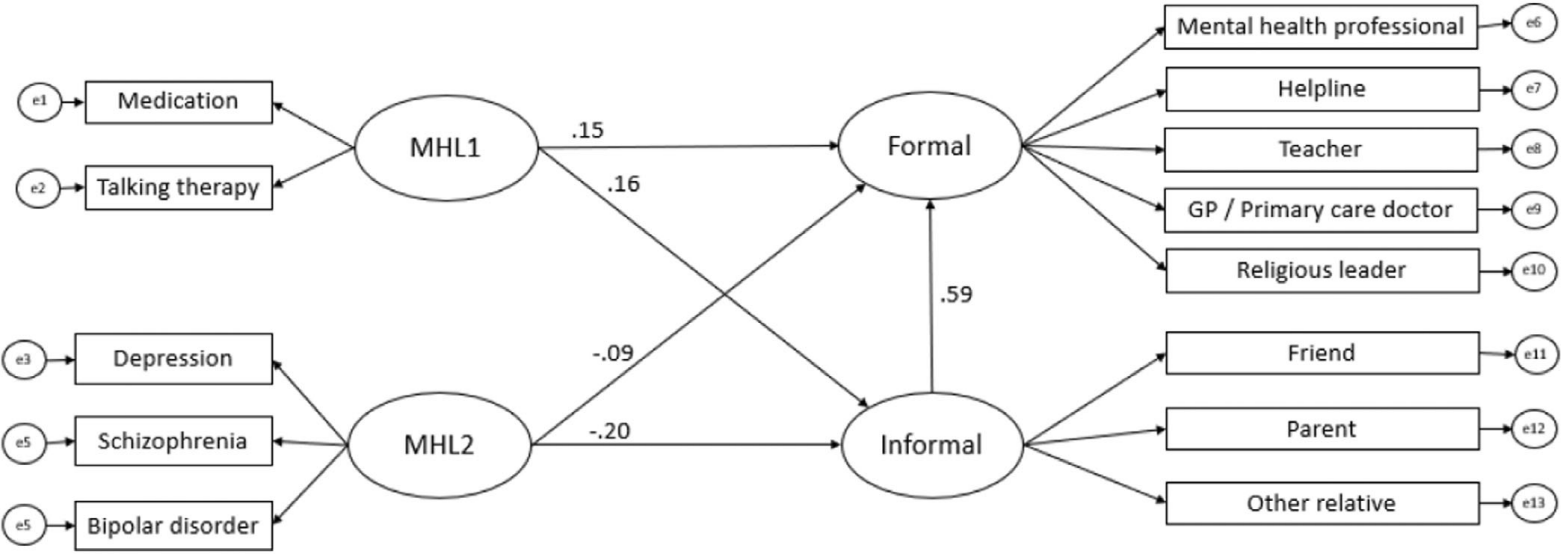
Final model assessing associations between mental health literacy (MHL) dimensions and formal and informal help‐seeking. Standardised estimates are displayed. MHL1 = Knowledge of treatment efficacy; MHL2 = Ability to identify mental health problems

Analysis testing the hypothesised direct and indirect association between dimensions of MHL, and forms of help‐seeking intention (Figure 1) were examined considering all covariates. Findings indicated a good model fit (χ^2^ (59) = 175.738, *p* < .001; RMSEA = .052 (90% Confidence Interval .043, .061); CFI = .947; TLI = .930; SRMR = .0415) and all estimates were theoretically meaningful (Table S3).

### Direct associations between dimensions of MHL and forms of help‐seeking

Results indicate a positive association between knowledge of treatment efficacy and informal help‐seeking intention (β = .154, *p* = .015) and formal help‐seeking intention (β = .158, *p* = .003). In other words, adolescents who reported higher levels of knowledge of treatment efficacy were more likely to report higher levels of formal and informal help‐seeking intentions. On the other hand, ability to identify a mental health problem was negatively associated with both informal (β = −.204, *p* = .012) and formal help‐seeking intentions (β = −.089, *p* = .049) indicating that adolescents who report higher ability to identify a mental health problem were less likely to report intentions to seek informal and formal help for mental health problems.

### Indirect association between MHL and help‐seeking intentions

Knowledge of treatment efficacy demonstrated a positive indirect association with formal help‐seeking intention via informal help‐seeking intention (β = .092, *p* = .017). Specifically, adolescents with higher knowledge of treatment efficacy were more likely to report higher intention to seek informal help, and those with higher informal help‐seeking intentions report a higher intention to seek formal help. Participants' ability to identify a mental health problem, on the other hand, had a negative indirect association with formal help‐seeking intention via informal help‐seeking intention (β = −.121, *p* = .011). Adolescents with a greater ability to recognise a mental health problem were more likely to report reduced intention to seek informal help, and those with lower intention to seek informal help were more likely to report reduced intention to seek formal help.

### Total effects of MHL on help‐seeking

The total effect of knowledge of treatment efficacy was positive for both informal (β = .154, *p* = .015) and formal help‐seeking intentions (β = .250, *p* = .003). This shows a strong association between knowledge of effective treatments and help‐seeking intentions. However, ability to recognise a mental health problem showed a negative association with both informal (β = −.204, *p* = .012) and formal help‐seeking intentions (β = −.210, *p* = .008), indicating that this form of knowledge is associated with lower help‐seeking intentions.

### Moderating effect of gender

Multigroup invariance analysis was used to evaluate any gender differences by estimating models simultaneously for both male and female participants, and by assessing unconstrained model fit, then adding constraints first to factor loadings, intercepts and path coefficients (Table S4). The fully constrained model was compared to the unconstrained model to assess for invariance. Changes in all fit indices were within acceptable parameters (ΔCFI = .005; ΔSRMR = .0003; ΔRMSEA = .005, Chen, 2007). Thus, invariance was supported, and gender was not identified as a moderator of MHL and help‐seeking intention in an adolescent sample.

## Discussion

We aimed to discern whether distinct dimensions of MHL are differentially associated with formal and informal help‐seeking intentions among adolescents, whether there is in an indirect effect of informal help‐seeking on formal help‐seeking, and whether gender moderates these associations. We demonstrate that while higher knowledge of treatment efficacy was associated with higher likelihood of reporting formal and informal help‐seeking intentions, ability to identify a mental health problem was associated with lower help‐seeking intention. Informal help‐seeking intention mediated the association between both forms of MHL and formal help‐seeking intention. Gender did not moderate the association between MHL and help‐seeking intention. To the best of our knowledge, this study is the first to identify that some dimensions of MHL, specifically adolescents' ability to discern what is, and what is not a mental health problem, are associated with lower likelihood of reporting intention to seek help.

Results of SEM analyses identified a mediating effect of informal help‐seeking intention, indicating that informal help‐seeking intention represents a potential mechanism by which MHL can affect formal help‐seeking. This is particularly important as research has noted that while adolescents are less likely to seek formal help (Radez et al., 2021), formal help‐seeking serves as a protective factor against worsening mental health (Neufeld et al., 2017). Therefore, it is important to develop strategies that encourage informal help‐seeking among adolescents, as this may influence formal help‐seeking intention.

We found that ability to identify a mental health problem was negatively associated with formal and informal help‐seeking intentions. MHL is frequently conceptualised by focussing on recognition of mental disorders (Mansfield, Patalay, & Humphrey, 2020). This may promote a psychiatric and biogenetic conceptualisation of mental health problems (Read, 2007), which has been demonstrated to be associated with erroneous beliefs about dangerousness and unpredictability of those experiencing a mental health problem (Kvaale, Haslam, & Gottdiener, 2013). Conceptualising MHL based on recognition of diagnostic labels may therefore lead to negative attitudes, such as stigma towards those experiencing mental health problems (Kinderman, Read, Moncrieff, & Bentall, 2013; Schomerus et al., 2012). For instance, evidence suggests that perceived stigma around these diagnoses may be why this dimension of MHL is associated with reduced help‐seeking intention (Gulliver et al., 2012). For this reason, it is important to note that both schizophrenia and bipolar disorder were included within the ‘ability to identify a mental health problem’ dimension in our study, both of which are known to receive high degrees of stigma within an adolescent population (DuPont‐Reyes, Villatoro, Phelan, Painter, & Link, 2020; Yoshioka, Reavley, MacKinnon, & Jorm, 2014). Therefore, the negative associations between ability to identify a mental health problem, and willingness to seek formal and informal help, may in part be due to the stigma towards such mental health conditions. Furthermore, research has noted that different aspects of stigma (e.g., personal and perceived stigma, or explicit and implicit stigma) may be differently associated with help‐seeking intention (Ma et al., 2022; Nearchou et al., 2018). There exists a complex relationship between MHL, stigma and help‐seeking, as a result, we cannot generalise the current findings to knowledge of other mental disorders, and future research should investigate if associations with help‐seeking differ by severity or typology of mental health conditions, and how discrete components of MHL may impact different forms of stigma towards mental ill health.

Previous research (Mansfield, Humphrey, & Patalay, 2020) has noted a correlation between MHL, when measured by the MAKS (Evans‐Lacko et al., 2010), and help‐seeking intention when measured by the GHSQ (Wilson et al., 2005), both of which were used in the current study. However, the association was ‘very small’ (Mansfield, Humphrey, & Patalay, 2020, p. 287). Based on our findings, we argue that this may be due to the differing associations that dimensions of MHL contained with the MAKS may have on help‐seeking intentions. Namely that increased ability to identify a mental health problem is associated with decreased help‐seeking intention, while knowledge of treatment efficacy is associated with increased intention.

In relation to gender differences, we found that the hypothesised associations between the MHL and help‐seeking were similar for boys and girls. This indicated that among our sample, gender did not moderate the associations between MHL constructs and formal or informal help‐seeking intention. However, our findings do align with research which has found limited effects of gender on MHL (Furnham, Annis, & Cleridou, 2014). As stigma is known to demonstrate gender differences and is a known deterrent to help‐seeking, future research should also investigate the role of stigma as a moderating factor in the relationship between MHL and help‐seeking intention.

### Limitations and future research

The results must be interpreted with an awareness of key limitations. First, we did not ask participants whether they had previously sought help for a mental health problem. Therefore, it is not possible to evaluate whether those who have experience of formal help‐seeking responded differently to service‐naïve counterparts. This may be a beneficial avenue for future research.

Second, while there is a growing awareness that young people are turning to the internet and social media for support (Pretorius, Chambers, & Coyle, 2015), this was not measured in the current study, and items in the General Help Seeking Questionnaire (Wilson et al., 2005), which refer to ‘helplines’ may be outmoded for adolescents in a contemporary context.

Third, we measured intended help‐seeking, not actual help‐seeking. While help‐seeking intention predicts help‐seeking behaviour (Wilson et al., 2005), behavioural intentions do not always lead to actual behaviour (Armitage & Conner, 2001). It is vital to consider factors such as self‐efficacy and availability of and access to mental health services, which are equally crucial for actual help‐seeking behaviour (Rimal, 2001). It may be useful for future work to assess longitudinally whether the dimensions of MHL identified in our study are associated with subsequent help‐seeking behaviour, as well as to look at broader sociocontextual factors such as availability of services. Additionally, like other studies, we examined association between MHL and help‐seeking intention and not behaviour, and as a result caution is needed in inferring causality with behaviour.

Fourth, our use of the MAKS (Evans‐Lacko et al., 2010) allowed us to identify that increased ability to identify a mental health problem was associated with decreased help‐seeking intention. It may be that mental health problems contained in the MAKS, such as schizophrenia are less familiar to adolescents, and may not have captured dimensions of MHL in which adolescents have greater knowledge. However, the aim of the current study was to capture discrete dimensions, rather than a broader overview of adolescent MHL. Additionally, the MAKS was not designed to capture all components of MHL, such as knowledge of how to seek help, and so all dimensions were not captured in the current study. Relatedly, the nature of our analysis means only 5 of the 12 items of the MAKS (i.e., those that were included in the two‐factor structure of MHL) were investigated for their associations with help‐seeking intention. Therefore, future research should investigate additional dimensions of MHL, including those relating to positive mental health and determine whether their impact on help‐seeking intentions is similar to the results we report here.

Finally, the cross‐sectional design allowed us to explore associations, but limits our ability to make causal interferences. Future studies should consider longitudinal and naturalistic experiments or intervention studies to examine causal pathways between discrete dimensions of MHL and both formal and informal help‐seeking intentions among adolescents.

## Conclusions

This article has demonstrated the importance of adopting a nuanced approach to the study of MHL and its relationship with help‐seeking intentions among adolescents. The current research extends previous work in adolescent MHL and help‐seeking by delineating specific dimensions of MHL which may be associated with the help‐seeking intention among adolescents. We identified that while knowledge of treatment efficacy is associated with higher likelihood of formal and informal help‐seeking; ability to identify a mental health problem was associated with lower likelihood of intentions to seek help. Findings demonstrate that some specific dimensions of MHL may be associated with lower likelihood of help‐seeking intention and therefore, should not be the sole focus of MHL interventions. Specifically, our results suggest that among adolescents, information about specific mental health problems are not sufficient to increase help‐seeking intentions, but rather, knowledge of effective treatments should be central to MHL‐based discussion to increase adolescents' willingness to seek help.

## Author contributions

CG, ES and AM conceived and developed the study. CG was responsible for study recruitment and data collection. CG had full access to all the data in the study and takes responsibility for the integrity of the data in the study and the accuracy of the data analysis. Statistical analysis was led by CG and ES. All authors participated in the interpretation of findings, contributed core ideas, and read and approved the final manuscript.

## Ethical information

The study was approved by University of Strathclyde, School of Education Ethics Committee. Informed consent was gained from all participants, parents and head teachers involved in the study.

## Supporting information


**Figure S1.** Final two factor model of Mental Health Literacy following CFA. MHL1 = Knowledge of treatment efficacy; MHL2 = Ability to identify a mental health problem.
**Figure S2.** Final two factor model of help‐seeking intention following CFA.
**Table S1.** Descriptive Statistics (*n* = 734).
**Table S2**. Correlation matrix for key variables.
**Table S3**. Direct, indirect and total effects of model including covariates.
**Table S4**. Direct, indirect and total effects of moderation model with constrained factor loadings, intercepts and regression weights.
