# Supplementary material for: Associations between dimensions of mental health literacy and adolescent help‐seeking intentions
Source: Child Adolesc Ment Health. 2022 Nov 14;28(3):385–92. doi: 10.1111/camh.12608 (PMC10947377; doi:10.1111/camh.12608)
Supplement: Supplementary file 1 — Figure S1. Final two factor model of Mental Health Literacy following CFA. MHL1 = Knowledge of treatment efficacy; MHL2 = Ability to identify a mental health problem. Figure S2. Final two factor model of help‐seeking intention following CFA. Table S1. Descriptive Statistics (n = 734). Table S2. Correlation matrix for key variables. Table S3. Direct, indirect and total effects of model including covariates. Table S4. Direct, indirect and total effects of moderation model with constrained factor loadings, intercepts and regression weights. [file CAMH-28-385-s001.docx]

**Supporting Information**

| **Table S1**: Descriptive Statistics (*n*=734) | | |
| --- | --- | --- |
| Variable | Mean (SD)/% | min-max info |
| *Gender*  Male  Female | 46.9%  52.1% | 0 = male  1 = female |
| Age | 14.23 (1.51) | 12-17 years |
| Subjective SES | 2.47 (0.85) | 1 (very well off) –  5 (not at all well off). |
| SIMD tercile | 1.83 (0.74) | 1 (most deprived) –  3 (least deprived) |
| *Ethnicity*  White  Other ethnicity | 88.4%  11.3% | 0 = White  1 = Other ethnicity |
| *Mental Health Literacy*  Knowledge of treatment efficacy | 7.58 (1.55) | 2 – 10 (higher scores = greater knowledge) |
| Ability to identify a mental health problem | 13.40 (1.98) | 3-15 (higher scores = greater knowledge) |
| *Help-seeking*  Informal | 14.36 (4.16) | 3 – 21 (higher scores = greater likelihood of seeking help) |
| Formal | 15.50 (6.43) | 5 – 35 (higher scores = greater likelihood of seeking help) |
| SD= standard deviation; SES= socioeconomic status; SIMD= Scottish Index of Multiple Deprivation | | |

| **Table S2:** Correlation matrix for key variables | | | | | | | | | | |
| --- | --- | --- | --- | --- | --- | --- | --- | --- | --- | --- |
| Variable | MHL1 | MHL2 | Informal | Formal | Gender | Age | Ethnicity | SES | SIMD |  |
| MHL1 | -- | .**193^**^** | **.094^*^** | **.113^**^** | .029 | **-.099^**^** | -.014 | .024 | -.014 |  |
| MHL2 |  | -- | **-.101^**^** | **-.134^**^** | .013 | **0.074^*^** | .037 | .049 | **-.080^*^** |  |
| Informal |  |  | -- | **.426^**^** | -.001 | .007 | -.027 | .015 | .044 |  |
| Formal |  |  |  | -- | .002 | .012 | .034 | -.007 | .048 |  |
| Gender |  |  |  |  | -- | .013 | .003 | -.010 | .070 |  |
| Age |  |  |  |  |  | -- | .008 | .001 | **.076^*^** |  |
| Ethnicity |  |  |  |  |  |  | -- | -.002 | .038 |  |
| SES |  |  |  |  |  |  |  | -- | **-.142^**^** |  |
| SIMD |  |  |  |  |  |  |  |  | -- |  |
| ** p <.01; * p <.05  MHL1: first MAKS subscale following CFA, knowledge of treatment efficacy; MHL2: second MAKS subscale following CFA, ability to identify a mental health problem. SIMD: measure of school neighbourhood deprivation  Maximum possible score for: informal help-seeking = 21; formal help-seeking = 35 | | | | | | | | | | |

| Table S3: Direct, indirect, and total effects of model including covariates | | | | | |  |
| --- | --- | --- | --- | --- | --- | --- |
| Direct Effects | Standardised  Est (SE) | | | | Unstandardised  Est (SE) |  |
| MHL1 -> Informal | **.154 (.070) *** | | | | **.074 (.047) *** |  |
| MHL1-> Formal | **.158 (.054) **** | | | | **.300 (.111) **** |  |
| MHL2 -> Informal | **-.204 (.054) *** | | | | **-.166 (.059) *** |  |
| MHL2 -> Formal | **-.089 (.058) *** | | | | **-.285 (.148) **** |  |
| Informal -> Informal | **.594 (.039) **** | | | | **2.335 (1.20) **** |  |
| Ethnicity -> Informal | -.008 (.027) | | | | .000 (.020) |  |
| Ethnicity -> Formal | **.040 (.023) *** | | | | .001 (.129) |  |
| Age -> Informal | .001 (.043) | | | | .000 (.010) |  |
| Age -> Formal | .018 (.038) | | | | .016 (.034) |  |
| Family SES -> Informal | .013 (.044) | | | | .005 (.019) |  |
| Family SES-> Formal | -.013 (.036) | | | | -.021 (.059) |  |
| School n’hood deprivation -> Informal | .049 (.041) | | | | .006 (.006) |  |
| School n’hood deprivation -> Formal | .006 (.038) | | | | .003 (.019) |  |
| Indirect Effects | Standardised | | |  | Unstandardised |  |
| MHL1 -> Informal -> Formal | **.092 (.042) *** | | | | **.174 (.092) *** |  |
| MHL2 -> Informal -> Formal | **-.121 (.033) *** | | | | **-.387 (.112) **** |  |
| Ethnicity -> Informal -> Formal | -.005 (.016) | | | | .000 (.050) |  |
| Age -> Informal -> Formal | .001 (.025) | | | | .001 (.023) |  |
| Family SES -> Informal -> Formal | .008 (.027) | | | | .012 (.043) |  |
| School n’hood deprivation-> Informal -> Formal | .029 (.024) | | | | .015 (.012) |  |
| Total Effects | | Standardised |  | | Unstandardised |  |
| MHL1 -> Informal | **.154 (.070) *** | | | | **.074 (.047) *** |  |
| MHL1 -> Formal | **.250 (.061) **** | | | | **.300 (.111) **** |  |
| MHL2 -> Informal | **-.204 (.054) *** | | | | **-.166 (.059) **** |  |
| MHL2 -> Formal | **-2.10 (.053) **** | | | | **-.285 (.148) **** |  |
| Ethnicity -> Informal | -.008 (.027) | | | | .000 (.020) |  |
| Ethnicity -> Formal | **.035 (.028) *** | | | | **.001 (.159) *** |  |
| Age -> Informal | .001 (.043) | | | | .000 (.010) |  |
| Age -> Formal | .019 (.042) | | | | .017 (.038) |  |
| Family SES -> Informal | .013 (.044) | | | | .005 (.019) |  |
| Family SES-> Formal | -.005 (.041) | | | | -.009 (.066) |  |
| School n’hood deprivation -> Informal | .049 (.041) | | | | .006 (.006) |  |
| School n’hood deprivation -> Formal | .035 (.042) | | | | .018 (.021) |  |
| *** p *<.*001; ** p < .01; *p < .05  MHL1 = Knowledge of treatment efficacy; MHL2 = Ability to identify mental health problems  Covariates include: age, ethnicity, family socioeconomic status and school neighbourhood deprivation | | | | | | |

| Table S4: Direct, indirect and total effects of moderation model with constrained factor loadings, intercepts and regression weights. | | | | | | | | | |
| --- | --- | --- | --- | --- | --- | --- | --- | --- | --- |
|  | Male (*n* =344) | | | | | Female (*n =* 382) | |  |  |
| Direct Effects | Standardised  Est (SE) | | | | Unstandardised  Est (SE) | Standardised  Est (SE) | Unstandardised  Est (SE) |  |  |
| MHL1 -> Informal | .145 (.065) * | | | | .069 (.040) * | .145 (.065) * | .069 (.040) * |  |  |
| MHL1-> Formal | .162 (.051) ** | | | | .302 (.109) ** | .162 (.051) ** | .302 (.109) ** |  |  |
| MHL2 -> Informal | -.209 (.052) ** | | | | -.172 (.057) ** | -.209 (.052) ** | -.172 (.057) ** |  |  |
| MHL2 -> Formal | -.094 (.048) * | | | | -.303 (.152) * | -.094 (.048) * | -.303 (.152) * |  |  |
| Informal -> Formal | .599 (.038) ** | | | | 2.358 (.625) ** | .599 (.038) ** | 2.358 (.625) ** |  |  |
| Indirect Effects | Standardised | | |  | Unstandardised | Standardised | Unstandardised |  |  |
| MHL1 -> Informal -> Formal | .087 (.039) * | | | | .162 (.084) * | .087 (.039) * | .162 (.084) * |  |  |
| MHL2 -> Informal -> Formal | -.125 (.031) ** | | | | -.405 (.104) ** | -.125 (.031) ** | .318 (.104) ** |  |  |
| Total Effects | | Standardised |  | | Unstandardised | Standardised | Unstandardised |  |  |
| MHL1 -> Informal | .145 (.065) * | | | | .069 (.040) * | .145 (.065) * | .069 (.040) * |  |  |
| MHL1 -> Formal | .249 (.057) ** | | | | .464 (.141) ** | .249 (.057) ** | .464 (.141) ** |  |  |
| MHL2 -> Informal | -.209 (.052) ** | | | | -.172 (.057) ** | -.209 (.052) ** | -.172 (.057) ** |  |  |
| MHL2 -> Formal | -.219 (.057) ** | | | | -.708(.177) ** | -.219 (.057) ** | -.708(.177) ** |  |  |
| *** p *<.*001; ** p < .01; *p < .05  MHL1 = Knowledge of treatment efficacy; MHL2 = Ability to identify mental health problems | | | | | | | | |  |

| 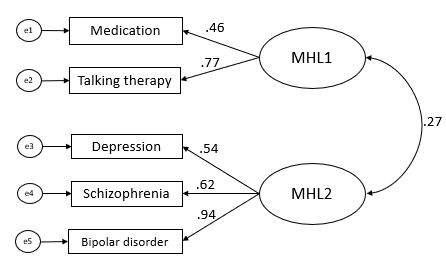 |
| --- |
| **Fig.S1**. Final two factor model of Mental Health Literacy following CFA.  MHL1 = Knowledge of treatment efficacy; MHL2 = Ability to identify a mental health problem |

| 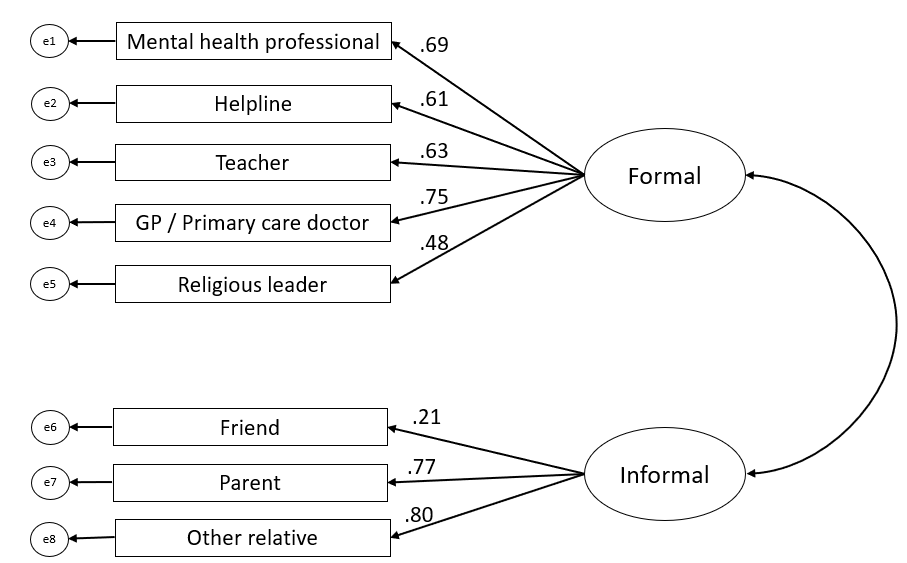 |
| --- |
| **Fig S2**. Final two factor model of help-seeking intention following CFA |

Definition of “mental health problems” used in the study:

“Mental health problems can influence how someone thinks, feels and behaves, and there are many different types of mental health problems. Mental health problems may mean people feel worried or unhappy or have difficulties with their thoughts, feelings and behaviour in ways that affect their everyday life.”
